# Similarities and Differences in the Pattern of Tau Hyperphosphorylation in Physiological and Pathological Conditions: Impacts on the Elaboration of Therapies to Prevent Tau Pathology

**DOI:** 10.3389/fneur.2020.607680

**Published:** 2021-01-07

**Authors:** Antoine Duquette, Camille Pernègre, Ariane Veilleux Carpentier, Nicole Leclerc

**Affiliations:** ^1^Research Center of the University of Montreal Hospital (CRCHUM), Montréal, QC, Canada; ^2^Département de Neurosciences, Faculty of Medicine, Université de Montréal, Montréal, QC, Canada

**Keywords:** tau protein, hyperphosphorylation, tauopathies, microtubules, Alzheimer's disease

## Abstract

Tau protein, a neuronal microtubule-associated protein, becomes hyperphosphorylated in several neurodegenerative diseases called tauopathies. Hyperphosphorylation of tau is correlated to its redistribution from the axon to the somato-dendritic compartment at early stages of tauopathies. Interestingly, tau hyperphosphorylation begins in different regions of the brain in each tauopathy. In some regions, both neurons and glial cells develop tau hyperphosphorylation. Tau hyperphosphorylation is also observed in physiological conditions such as hibernation and brain development. In the first section of present article, we will review the spatiotemporal and cellular distribution of hyperphosphorylated tau in the most frequent tauopathies. In the second section, we will compare the pattern of tau hyperphosphorylation in physiological and pathological conditions and discuss the sites that could play a pivotal role in the conversion of non-toxic to toxic forms of hyperphosphorylated tau. Furthermore, we will discuss the role of hyperphosphorylated tau in physiological and pathological conditions and the fact that tau hyperphosphorylation is reversible in physiological conditions but not in a pathological ones. In the third section, we will speculate how the differences and similarities between hyperphosphorylated tau in physiological and pathological conditions could impact the elaboration of therapies to prevent tau pathology. In the fourth section, the different therapeutic approaches using tau as a direct or indirect therapeutic target will be presented.

## Tau Protein and Its Role in Tauopathies

**Figure 1:**
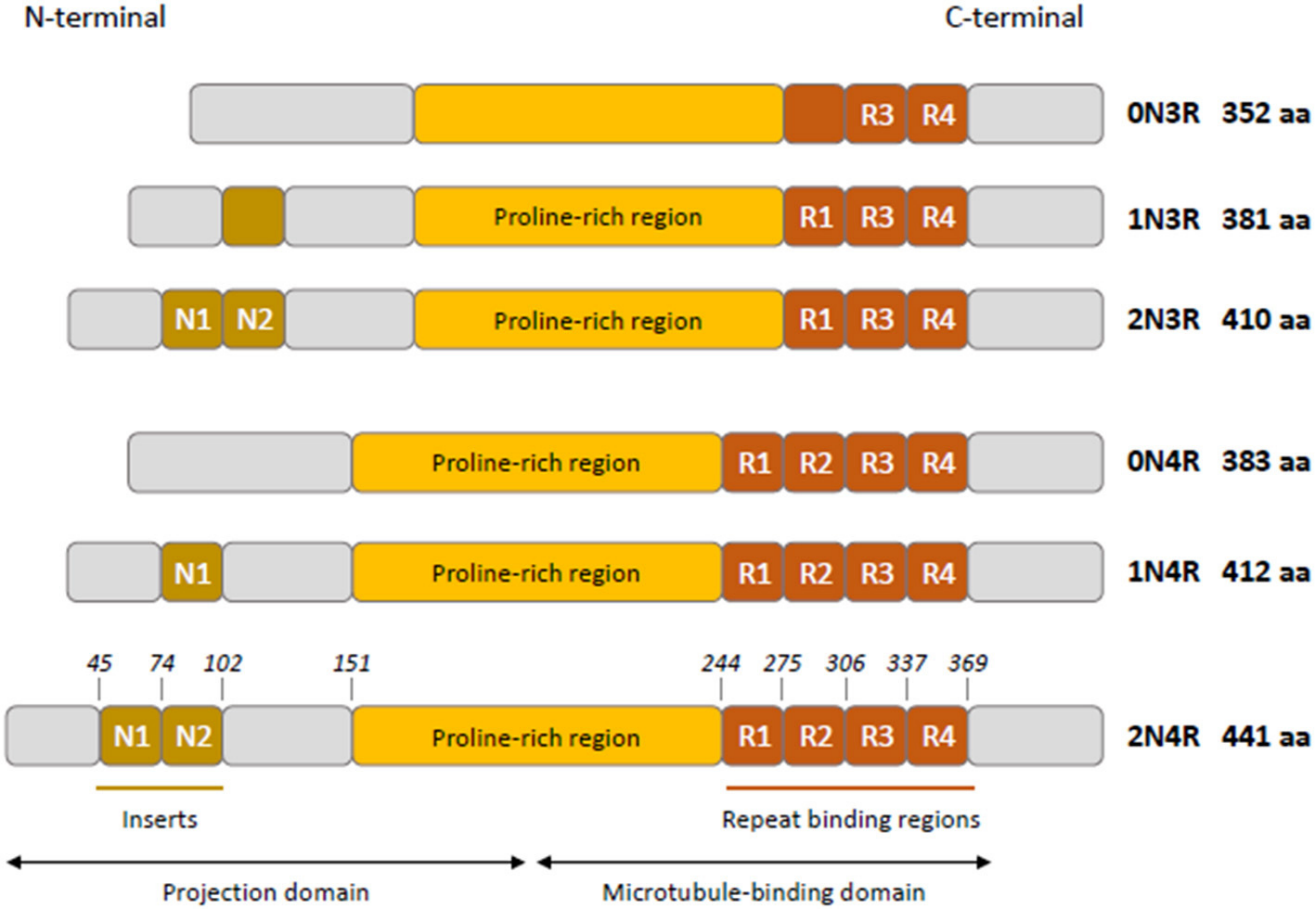
Schematic representation of tau isoforms in human adult brain. Tau isoforms (0N3R, 1N3R, 2N3R, 0N4R, 1N4R, 2N4R) are generated by alternative splicing of exons 2, 3, and 10. Two inserts N1 and N2 (brown boxes) near the N-terminus of tau are produced by exons 2 and 3, respectively. Absence of N1 and N2 gives rise to 0N isoforms, whereas inclusion of N1 produces 1N isoforms and inclusion of both N1 and N2 results in 2N isoforms. The proline-rich region is indicated in yellow. R1–R4 (orange boxes) represent the four microtubule- binding repeats, R2 being encoded by exon 10. The exclusion or inclusion of exon 10 results in isoforms with three or four microtubule-binding repeats named 3R and 4R isoforms.

Tau is mainly a neuronal microtubule-associated protein encoded by the *MAPT* gene on human chromosome 17q21 (1). The *MAPT* gene has 16 exons; three of them are subject to alternative splicing (exons 2, 3, and 10), giving rise to the six isoforms in human adult brain (2, 3). Tau has four functional domains: an amino-terminal projection domain (up to two amino-terminal inserts), a proline-rich region, the microtubule-binding domain (three or four microtubule-binding repeats), and the carboxy-terminal region (Figure 1) (4). Alternative splicing of exon 10 produces isoforms with either three or four microtubule-binding repeats named 3R and 4R isoforms (2). Tau is mainly localized to the axon and plays a role in maintaining neuronal integrity and axonal transport by regulating microtubule assembly and dynamics (5, 6). Its presence in dendrites and nuclei of neurons indicates its involvement in synaptic signaling and genome stability (7). Tau is expressed at very low levels in astrocytes and oligodendrocytes where its role in microtubule assembly and stability remains poorly characterized (7–9).

Tau undergoes several post-translational modifications in physiological conditions; phosphorylation being the predominant modification. Most of these modifications impact tau function and contribute to the heterogeneity of tau forms found in developing and adult brain (7). In neurodegenerative diseases called tauopathies, tau becomes hyperphosphorylated destabilizing its interaction with microtubules, accumulates and self-aggregates in insoluble filaments (7, 10). This aggregation of tau is correlated to neurodegeneration (11, 12). The exact role of other post-translational modifications (glycation, O-GlcNAcylation, nitration, acetylation, methylation, SUMOylation, ubiquitylation, oxidation, and truncation) in aggregation is increasingly investigated (12, 13).

In Alzheimer's disease (AD), both tau and amyloid-beta (Aβ) pathologies are landmarks of the disease whereas in primary tauopathies, tau pathology is the main neuropathological landmark (14–17). Frontotemporal lobar degeneration with predominant tau pathology (FTLD-tau) comprises primary tauopathies affecting the frontal and temporal lobes. This classification encompasses Pick's disease (PiD), progressive supranuclear palsy (PSP), corticobasal degeneration (CBD), globular glial tauopathies (GGT), and argyrophilic grain disease (AGD). The distribution of cellular tau (neuronal, glial, or mixed), the conformation of tau filaments, and the spatiotemporal pattern distinguish these tauopathies (7, 15). The diagnosis of tauopathies can be challenging. In the aging brain, neurodegenerative diseases frequently coexist and overlapping pathological features are common (14, 18). The clinical phenotype depends on the brain regions affected and not necessarily on the molecular changes occurring at the cellular levels. Thus, while some phenotypes are more frequent in a specific disease, there is a weak correlation between the clinical symptoms and the disease involved.

Tau contains 85 phosphorylation sites. Ten sites are phosphorylated in human control brain whereas in AD brain, 55 sites become phosphorylated (19, 20). Proline directed and non-proline directed kinases were shown to contribute to tau hyperphosphorylation. Phosphatase 2A (PP2A) is the main tau phosphatase and the decrease of its activity in AD brain is believed to contribute to tau hyperphosphorylation (21, 22). Tau hyperphosphorylation is correlated to its redistribution in the somato-dendritic compartment observed at early stages of tau pathology (12). Tau hyperphosphorylation is also observed in physiological conditions. Both in brain development and hibernation, tau is hyperphosphorylated. In the present article, we will firstly review the spatio-temporal and cellular distribution of hyperphosphorylated tau in tauopathies. Then we will compare the pattern of tau hyperphosphorylation in tauopathies and physiological conditions. Lastly, we will discuss how the similarities and differences of tau hyperphosphorylation in tauopathies and physiological conditions impact the elaboration of therapies to prevent tau hyperphosphorylation and aggregation.

## Section (1) the Spatiotemporal and Cellular Distribution of Hyperphosphorylated Tau Varies in Different Tauopathies

Tau pathology is found in several neurodegenerative diseases that are listed in Tables 1, 2. In the present section, we will review the spatiotemporal pattern of the propagation of tau pathology and the cellular distribution of hyperphosphorylated tau in AD and the three most studied primary tauopathies: PSP, CBD, and PID. Most of the PSP, CBD, and PID cases are sporadic frontotemporal lobar degeneration (FTLD) with tau-immunoreactive inclusions (FTLD-tau). However, some cases affected by one of these diseases present a mutation in the *MAPT* gene. Forrest and al found that 59% of their *MAPT* cases could be pathologically subclassified as other sporadic tauopathies including PSP, CBD, and PiD (23). PSP and CBD are 4R-tauopathies given that tau aggregates are solely composed of 4R isoforms in these diseases whereas PID is a 3R-tauopathy because of its tau aggregates composed of 3R isoforms. Each disease is characterized by unique type of tau filaments (24). In recent studies, the atomic structure of tau filaments in AD, PID, and CBD was analyzed by Cryo-EM (25–27). In each of these diseases, the extent of the ordered cores of the different folds varies.

**Table 1:** Pathological characteristics of tauopathies.

**Tauopathy**	**Neuronal or glial**	**Tau isoform**	**Tau filament structure**	**Main pathologic features of tau inclusions**	**Other microscopic pathological anomalies**
Pick's disease	Neuronal > glial	3R	15–18 nm diameter straight filaments and 22–24 nm twisted	Pick bodies (round neuronal inclusions) Ramified astrocytes	Ballooned neurons (possibly secondary to tau pathology)
Progressive supranuclear palsy	Both	4R	15–18 nm diameter straight filaments	NFT Pretangles Neuropil threads Tufted astrocytes Coiled bodies	
Corticobasal degeneration	Both	4R	24 nm diameter twisted filaments	Neuropil threads Pretangles Astrocytic plaques Coiled bodies	Ballooned neurons (possibly secondary to tau pathology)
Chronic traumatic encephalopathy	Both	3R + 4R	20–25 diameter paired helical filaments	NFT Pretangles Astrocytic tangles	TDP-43 inclusions (unknown if triggered by tau or involved in tau accumulation)
Globular glial tauopathies	Both	4R	Paired helical filaments and straight filaments	Spherical neuronal cytoplasmic inclusions Globular oligodendroglial and astroglial inclusions	
Primary age-related tauopathy	Neuronal > Glial	3R + 4R	Paired helical filaments and straight filaments	Coiled bodies (unknown if related to PART or normal aging)	
Argyrophilic grain disease		4R	9–18 nm diameter straight filaments and 25 nm filaments forming compact bundles (grains)	Argyrophilic grains Pretangles Coiled bodies Diffuse granular astrocytic immunoreactivity	Ballooned neurons (possibly secondary to tau pathology)
Alzheimer's disease	Neuronal	3R + 4R	8–20 nm diameter paired helical filaments 15 nm diameter straight filaments	NFT Neuropil threads	Amyloid-beta plaques (primary neurodegenerative process with intricate relation with tau toxicity)

*NFT, neurofibrillary tangles*.

**Table 2:** Spatiotemporal pattern of accumulation of tauopathies.

**Tauopathy**	**Spatiotemporal pattern of neuronal tau accumulation**			**Main clinical phenotypes**
	**Early or pre-clinical**	**Mild stage**	**Late-stage**	
Pick's disease	Limbic system Neocortical frontotemporal cortex	Basal ganglia Brainstem Noradrenergic/serotonergic nuclei Dentate nucleus Sensory tract of the spinal cord	Pre-motor cortex Pre-cerebellar nuclei Primary visual cortex	bvFTD nfaPPA
Progressive supranuclear palsy	Globus pallidus Substantia nigra Subthalamic nucleus	Brainstem, cerebellum (dentate nucleus), posterior frontal lobe	Association cortices	PSP-RS PSP-P PSP-CBS bvFTD nfaPPA
Corticobasal degeneration	Anterior frontal cortex Basal ganglia	Posterior frontal and parietal cortices	Higher tau burden in same regions as early/mild stage Midbrain and pons	CBS bvFTD nfaPPA RS
Chronic traumatic encephalopathy	Depth of sulci in frontal lobe Nucleus basalis Meynert Locus coeruleus	Superficial cortical layers Nucleus basalis Meynert Locus coeruleus	Amygdala Hippocampus Temporal, parietal and insular cortices Diencephalon Brainstem Spinal cord	Cognitive dysfunction Emotional dysregulation Behavior change Motor disturbance
Globular glial tauopathies	(Unknown temporal pattern) Frontal and temporal lobes Motor cortex Corticospinal tracts			bvFTD bvFTD + MND MND PSP-RS CBS
Primary age-related tauopathy	Transentorhinal region	Limbic structures (entorhinal and hippocampus)	Rarely neocortex is affected	Amnestic mild cognitive changes
Argyrophilic grain disease	Ambient gyrus and CA1 of hippocampus	Amygdala Medial temporal lobe	Insular cortex Anterior cingulum Nucleus accumbens Septal nuclei Hypothalamus Gyri recti	Amnestic mild cognitive changes Behavior and psychiatric symptoms bvFTD
Alzheimer's disease	Transentorhinal region	Limbic structures (entorhinal and hippocampus)	Isocortical	From amnestic cognitive changes to dementia

*bvFTD, behavioral variant of frontotemporal dementia; nfaPPA, non-fluent-agrammatic variant of primary progressive aphasia; CBS, corticobasal syndrome; RS, Richardson syndrome; PSP, progressive supranuclear palsy; MND, motor neuron disease*.

## Alzheimer's Disease (AD)

AD is considered a secondary tauopathy because tau pathology is not the sole neuropathological landmark of the disease. Extracellular Aβ plaques are found in AD and are believed to trigger the disease whereas tau pathology would contribute to the progression of the disease by playing a central role in Aβ toxicity (9, 28, 29). In AD, tau pathology is predominant in neurons. Tau filaments are composed of 3R and 4R isoforms and formed neurofibrillary tangles (NFTs). Six stages (Braak stages) of tau pathology can be observed in AD (30). NFTs are first detected in the transentorhinal region and then extend to the entorhinal and hippocampus at stages I and II. At stages III and IV, NFTs propagate to the neocortical high-order associative areas. In stages V and VI, primary and secondary fields of the neocortex present NFTs. The extent of cognitive decline usually correlates with the accumulation of neocortical tau (31).

## Progressive Supranuclear Palsy (PSP)

PSP is an adult-onset disorder that presents cognitive impairment associated with behavioral changes, akinetic rigid syndrome, and prominent oculomotor dysfunction (32). The most prevalent clinical form is Richardson syndrome. The tau filaments that form NFTs are exclusively composed of 4R isoforms (9). Tau aggregates are also found in glial cells. Tufted astrocytes containing 4R tau aggregates are a specific neuropathological landmark of PSP (9). In oligodendrocytes, coiled bodies composed of 4R tau isoforms are also observed (9, 33). The pattern of tau pathology propagation differs for each cell type (neurons, astrocytes, or oligodendrocytes). Kovacs et al. have divided into six stages the pattern of tau pathology in the Richardson syndrome (34). NFTs firstly begin in the globus pallidus, substantia nigra, and subthalamic nucleus and secondly spread to the brainstem. Thirdly, NFTs are observed in the striatum, dentate nucleus and amygdala. Fourthly, NFTs are noted in the frontal lobe. In the fifth and sixth steps, NFTs develop in the parietal, temporal, and occipital lobes (34, 35). Interestingly, astroglial and oligodendroglial tau pathologies precede neuronal tau pathology in some brain regions. The oligodendroglial coiled bodies are detected in the globus pallidus at early stages of the disease before NFTs are observed in this region (34). Similarly, the aggregates of tau in astrocytes develop in the striatum before NFTs are noted. In the subsequent steps, astrocytic and oligodendroglial tau pathologies follow a distinct pattern of propagation for finally reaching the neocortex.

## Corticobasal Degeneration (CBD)

CBD is a tauopathy known for its asymmetric cortical atrophy (36, 37). Corticobasal syndrome (CBS) is the main clinical phenotype and comprise of asymmetric cortical signs (apraxia, hemineglect, alien limb, aphasia) and asymmetric parkinsonism or dystonia (18). In most neurons, tau staining is diffuse or granular resembling a pre-tangle stage. Both neuronal and glial thread-like tau-positive structures in the white and gray matter are observed in CBD. Tau-positive astrocytic plaques are the major neuropathological lesions of CBD. Neuronal loss is observed in focal cortical regions and in the substantia nigra (38). In CBD, the accumulation of tau follows a rostrocaudal gradient (37). It usually starts in the anterior frontal cortex with a higher ratio of astrocytic plaques compared to neuronal tau pathology. It evolves posteriorly and caudally with accumulation in the parietal cortex and brainstem structures. With the progression of the disease, neuronal tau burden increases, and there is an inversion of the ratio, with neuronal tau pathology being more prevalent than astrocytic plaques (37).

## Pick's Disease (PiD)

PiD is the sole tauopathy presenting neuronal inclusions uniquely composed of 3R isoforms (39). Symptoms and signs are commonly related to the affected frontal lobe and comprise of changes in behavior and personality, apathy, or progressive non-fluent aphasia. Pick bodies are fibrillar intra-neuronal spherical inclusions composed of 3R tau isoforms (9). The propagation of tau pathology occurs in 4 sequential steps. Firstly, tau pathology begins in the dentate gyrus of the hippocampus, the frontotemporal neocortical regions and the angular gyrus. Secondly, tau accumulates in the basal ganglia, the brainstem's noradrenergic and serotonergic nuclei, the dentate nucleus, and the sensory tract of the spinal cord. Thirdly, tau pathology is found in the pre-motor cortex and pre-cerebellar nuclei, and fourthly, in the primary visual cortex (39). Other tau pathologic features include ramified astrocytes and globular oligodendroglial coiled inclusions. However, it remains unclear if tau pathology in glial cells precedes neuronal tau pathology as noted in PSP.

### Pattern of Tau Phosphorylation in Tauopathies

In a recent study using mass spectrometry (MS), 55 phosphorylated sites were identified in two cohorts of AD (20). The highest frequency of phosphorylation was noted in the proline-rich region between the amino acids (a.a.) 195–209 and a.a. 212–224 and in the C-terminus in a.a. 396–406. Interestingly, insoluble tau aggregates were found in controls. High frequency phosphorylation sites in control insoluble tau were identified as threonine (T)181 and T231 and lower frequency of phosphorylation at serine (S)198, S199, S202, and T205 were observed in 20–40% of controls indicating that these sites could represent early stages of the disease. These results fit with previous studies where it was reported that T231 and S202/Thr205 were phosphorylated at early stages of AD (40–44). At intermediate stages of AD, 6 additional sites (S199, S202, T212, T217, S237, and S262) were identified in the proline-rich region and one in the C-terminal at S396 (20). The number of phosphorylation sites increased as the disease progresses to reach 55 at late stages indicating that the number of phosphorylation sites is associated with disease stages. Interestingly, no difference of phosphorylation of tau present in the soluble fraction was found between AD and controls as noted by others (20, 45). Tau found in the low molecular weight (monomers) and in the sarkosyl-soluble fractions (monomers) was seed incompetent while tau found in the high molecular weight (oligomers) and in the sarkosyl-insoluble fractions (fibrils) was seed competent.

Sixteen sites phosphorylated in AD were detected in PSP (46). The pattern of tau hyperphosphorylation has not been investigated at different stages of PSP. However, S202/T205 and T231, early sites in AD, are also phosphorylated in PSP (46, 47). As mentioned for PSP, the phosphorylation sites investigated until now for CBD and PiD are also found in AD (38, 39, 48, 49). In contrast to AD and PSP, some studies reported that there was no increase in tau phosphorylation at S262/S356 in PiD (48, 49). This was contradicted in another study (39). However, in a recent study where Cryo-EM was used to analyze the atomic structures of tau filaments in PiD, no phosphorylation at S262 or S356 was found (26).

## Section (II) Comparison of the Pattern of Tau Hyperphosphorylation in Physiological and Pathological Conditions

In both brain development and hibernation, hyperphosphorylated tau is present. Three important points will be compared between these physiological conditions and tauopathies in the present section (1) the pattern of tau hyperphosphorylation; (2) the role of hyperphosphorylated tau; and (3) the reversibility of tau hyperphosphorylation during brain development and hibernation but not in tauopathies.

### Pattern of Tau Hyperphosphorylation During Brain Development

Several groups reported that tau is hyperphosphorylated during fetal and post-natal brain development of rodents at sites similar to those observed in AD and primary tauopathies (50–54). This hyperphosphorylation was not correlated to neuronal cell death as noted in tauopathies. Few studies reported that tau is also hyperphosphorylated in human fetal brain (55, 56). However, the most limiting factor of these studies was the small number of individuals included in their analysis. In a recent study, tau phosphorylation was examined in 20 human fetal brains aged from 14 to 38 post-conceptional weeks and compared to that in tauopathies (AD, PSP, CBD, FTLD-tau), using a combination of immunohistochemistry and immunoblotting analysis (57). The authors showed that tau in human fetal brain is highly phosphorylated but its phosphorylation pattern differs from that of AD and primary tauopathies. Some sites such as S202, S214, and S396/S404 were strongly positive in a high percentage of cases as noted in AD and primary tauopathies. Other sites such as T231 found in high frequency at early stages of AD and S202/T205, less frequently phosphorylated at early stages of AD, were phospshorylated in a very low number of cases (20). All fetal brains were negative to S409, a site highly phosphorylated in AD. Interestingly, in this recent study, phospho-tau positive aggregates were found in fetal human brain. These aggregates were positive to T214 and weakly immunoreactive to the antibodies CP13 (S202) and PHF-1 (S396/S404). These aggregates apparently non-toxic were negative for thioflavin S staining indicating that they were seed-incompetent. Interestingly, in AD brain, S214, S396, and S404 were only found in seed competent tau fractions whereas S202 was found in seed incompent fractions (20). From the above observations, one can speculate that S202 could be one of the pivotal sites in preventing tau seeding activity.

The above studies revealed that in fetal brain, human tau is hyperphosphorylated at multiple epitopes, some of them overlapping with those seen in tauopathies. Human fetal tau appears to be able to form aggregates that are not seed competent. The function of such aggregates remains to be determined. All together, the above observations indicate that it is not hyperphosphorylation *per se* that is toxic but rather the phosphorylation of specific residues and/or the cellular context (development vs. aging) that seem to dictate the toxicity of tau. Further studies are needed to confirm this using more accurate methods such as MS to obtain the full picture of tau phosphorylation sites in fetal brain.

None of the studies mentioned above examined the distinct phosphorylation pattern of each tau isoform. This is an important point to consider since the pattern of tau isoforms is differently regulated in fetal and adult human brains. Only the smallest tau isoform is present in fetal brain whereas all 3R and 4R tau isoforms are found in the adult brain at a ratio of 1:1 (2, 55, 58–60). In rodents, a switch from 3R to 4R is observed during the development resulting in the sole presence of 4R isoforms in adult brain (55, 58, 60, 61). Interestingly, the developmental switch of tau isoforms and the changes of their phosphorylation occur 2–3 weeks postnatally, corresponding to the critical period of neuronal plasticity (62, 63). The 4R tau isoforms present the strongest microtubule-binding, -assembling and -stabilizing abilities and therefore a decrease of their phosphorylation is believed to increase their binding to microtubules, a crucial step for stabilizing the novel established connections in developing brain (64–66).

The difference of phosphorylation between tau isoforms remains poorly characterized. The distinct phosphorylation profile of tau isorforms was recently examined *in vivo* in mouse brain (67). This study, using a combination of the Phostag technology and 3R-/4R-specific antibodies, was the first one to report the differences between 3R and 4R phosphorylation *in vivo* although the phosphorylation sites that differ between 3R and 4R were not identified. During mouse brain development, 3R isoforms that were highly phosphorylated are replaced by the 4R isoforms presenting lower phosphorylation levels than 3R. The phosphorylation levels of both 3R and 4R isoforms decreased during brain development, the dephosphorylation of 4R being less important than that of 3R. A similar pattern of phosphorylation was observed for human 3R and 4R isoforms in knock-in mice indicating that in human brain, 3R and 4R isoforms could also display different profiles of phosphorylation. One has to take into consideration all these observations to compare the phosphorylation state of tau during brain development with that observed in tauopathies for the identification of the sites linked to tau toxicity.

#### Tau Hyperphosphorylation Associated With Axonal Outgrowth and the Establishment of Synaptic Contacts in Brain Development

During brain development, tau hyperphosphorylation is correlated to the growth of the axon and its suppression results in a reduced axonal length (68, 69). In developing Drosophila, tau was shown to be necessary for formation of synaptic contacts (70). Hyperphosphorylation of tau is believed to occur for decreasing microtubule stability and thereby allowing axonal growth in developing brain (71–73). Interestingly, axonal and dendritic sprouting was observed in AD indicating that neurons might attempt to re-growth their axon and re-establish synaptic contacts (74, 75). Tau hyperphosphorylation at early stages of tauopathies could be part of global reaction intended for re-starting developmental programs. Consistent with this, several studies have reported that markers of cell cycle are increased in AD brain (76). Interestingly, recent studies have shown that tau could be involved in cell cycle (77).

### Pattern of Tau Hyperphosphorylation in Hibernating Animals

Hibernation is a biological and adaptive process used by several mammalian species to survive in inhospitable environmental conditions. It is a powerful physiological strategy to restrict energy expenditure and compensate for periodically limited energy supply (78, 79). During hibernation, animals enter a hypometabolic state, called torpor, characterized by extreme changes: a blood-flow reduction, a decrease in brain and body temperature and an immune and metabolically depressed state (79–81). In the torpor state, the metabolic rate can be as low as 5% of that in normal euthermic state (81). Multiple periods of torpor lasting 3–4 days are periodically interrupted by short arousal periods of activity (usually <1 day) in which the animals return to euthermia and normal metabolism, blood flow and body temperature (80, 81). The accumulation of hyperphosphorylated Tau at S/T residues has been reported in several obligate and facultative hibernator species, as Arctic ground squirrels or Syrian hamsters during torpor (82–86). Hyperphosphorylation of tau were also found in neurons of hibernating European ground squirrels but totally disappeared as tau phosphorylation was fully reversed (82). Although the pattern of hyperphosphorylated tau is similar to the one in AD it appears to be non-toxic as no apparent neuronal damages were observed (79). Five phosphorylation sites (T181, S199, S202, T231, and S404) detected in hibernating animals were also found in tau seed incompetent fractions isolated from AD brain and another five sites (T205, S212, T214, S262, and S396) were found in competent tau seed competent fractions (20). This could indicate that in this physiological context, the phosphorylation sites preventing tau seeding activity override the effects of the sites favoring seeding.

In hibernating animals, hyperphosphorylation of tau is linked to synaptic deafferentation (79). In hibernating golden hamster, a regression of the dendritic spines on apical dendrites but not on basal dendrites of hippocampal neurons was noted (87). Interestingly, these changes were not linked to memory impairment. To explain this, the authors proposed that only unstable spines were removed whereas stable spines were spared.

#### Tau Hyperphosphorylation Associated With Protective Mechanisms in Hibernating Animals

Torpor in hibernating animals shares several abnormalities observed in AD such as same populations of neurons developing tau hyperphosphorylation, alterations of synaptic connections and cognitive impairment. The hibernation is a process requiring several biological mechanisms, which permit the regulation and adaption of the neural system (88). During torpor, neuronal structural changes have been reported, such as changes in dendritic spines and synaptic connections, in the Golgi apparatus morphology and alterations of microglial cells (89–93). Interestingly, all these changes are reversible. This very fast transition from torpor to arousal requires different remodeling of the brain such as reorganization of membranous organelles and synaptic formation (82, 93–95). In a recent study, the global metabolic changes in Syrian hamster brain during hibernation were analyzed (96). They have identified significant differences in more than 300 metabolites, providing new insights on adaptive and neuroprotective brain processes that are occurring during hibernation and arousal. For example, an increase of brain cryoprotectants such as theitol were increased during torpor whereas an increase of neuroprotective agents such as L-carnitine and acylcarnitine proposed as therapy for AD were elevated during arousal (97, 98). All these changes allow neurons to resist to damages upon cerebral ischemia during torpor and rapid reperfusion during arousal.

Hypometabolism is observed in hibernating animals as well as in AD and primary tauopathies. Some fluorodeoxyglucose positron emission tomography (PET) studies have detected hypometabolic states in isocortical brain areas of patients at preclinical stages of AD and patients with probable AD (subjects at risk) (99, 100). These abnormally low rates of cerebral glucose metabolism occur several decades before the possible onset of dementia and seem to predict cognitive decline (101, 102). Siegfried Hoyer was the first one to propose that hypometabolic states could eventually lead to AD pathology (103–106). In hibernating animals, the hypometabolic state corresponds to a reduction of energy supply and requirements, low neuronal activity and regression of synaptic connections. The neuronal activity is also drastically reduced (107–112). Some groups reported with electroencephalography measurements that almost no brain activity is present in the hibernating brain (110, 113, 114).

It was proposed that tau would play a role in regulating neuronal activity during the hypometabolic state. Indeed, the localization of tau at the synapse and its interaction with post-synaptic proteins modulating the insertion of receptors, in particular AMPA and NMDA receptors, at the plasma membrane make it a plausible player in regulating neuronal activity (115). Hyperexcitability is noted during cooling phase of hibernation as observed at early stages of AD (116, 117). Tau was shown to contribute to hyperexcitability in mouse models of epilepsy (118–120). Its suppression could abolish hyperexcitability in these models (119). The tau mutation V337M linked to frontotemporal dementia was reported to increase neuronal activity by altering the axonal initial segment plasticity (121). Bin1, a protein involved in AD pathogenesis, was shown to regulate tau-dependent hyperexcitability in hippocampal neurons (122). Lastly, two tau mutations, Δ280 and A152T, were reported to induce hypoexcitability and hyperexcitability, respectively, in neuronal cultures (123). Collectively, the above observations strongly support the contribution of tau in the regulation of neuronal activity in physiological and pathological conditions. Hyperphosphorylated tau in AD has recently been suggested to represent some compensatory response to suppress excitatory/inhibitory imbalance at initial stages of the disease. If this imbalance lasts too long, such as under conditions of uncontrolled prolonged hypometabolism, it may become pathological triggering a cascade of events leading to neurodegeneration. All together, the above observations indicate that the increase of tau phosphorylation at early stages of AD could be a physiological reaction to a reduced brain metabolic rate that is required for modulating neuronal activity.

### Tau Hyperphosphorylation Is Reversible During Brain Development and Hibernation

In humans, the hyperphosphorylation and aggregation of tau were frequently detected under the age of 30 in a systematic survey of more than 2 300 non-selected autoptic cases aged between 1 and 100 years (124, 125). The authors suggested that this might be the initial step of tauopathies. Moreover, in 2010, the National Institute on Aging and the Alzheimer's Association convened an international workgroup and regrouped some evidence suggesting that the pathological process begins at least years or decades before the onset of clinical impairment (126). These observations indicate that tau hyperphosphorylation is non-reversible in human adult brain leading to neurodegeneration. This fits with the decrease of phosphatase activity in AD brain (21, 127, 128).

During brain development and hibernation, tau hyperphosphorylation is transient and reversible. The highly phosphorylated tau is dephosphorylated around birth and during post-natal brain development to remain less phosphorylated in normal adult brain (51, 56). The role of decreasing tau phosphorylation during brain development is most likely to increase its binding to microtubules (71–73). In most studies, the hyperphosphorylation of Tau during torpor has been shown to be rapidly and fully reversed after arousal in hibernating animals (79, 82, 85, 86). However, a group reported that in Arctic ground squirrel, some sites were dephosphorylated in arousal animals while others remained phosphorylated, indicating a reversible phosphorylation at selective sites (84). Similarly, tau hyperphosphorylation was shown to be partially reversed in a model inducing a hypothermic torpor-like state (129). Although hypothermia could contribute to tau hyperphosphorylation during hibernation, it was clearly demonstrated that it was not sufficient and that tau phosphorylation is regulated by other mechanisms that remain to be identified.

As mentioned above, during hibernation, multiple periods of torpor lasting few days are periodically interrupted by short arousal periods of activity lasting hours in which the animals return to euthermia and normal metabolism, blood flow and body temperature (80, 81, 130, 131). The reasons for the repeated arousals are still not entirely clear, but it is believed that they allow repair of neuronal damages caused by prolonged hypometabolism and brain inactivity. Interestingly, aged black bears show permanent accumulation of hyperphosphorylated tau at a pretangle-like stage (132). This might be explained by the fact that their hibernation is a continuous torpor period, which is not interrupted by spontaneous arousal. Collectively, the above observations in hibernating animals indicate that tau hyperphosphorylation could be part of neuroprotective reaction but if it is not interrupted by normal periods of tau phosphorylation as noted during arousal in hibernation, it could become permanent making neurons more vulnerable to insults and neurodegeneration as noted in tauopathies.

## Section (III) Implications of Similarities and Differences in Tau Hyperphosphorylation Between Physiological Conditions and Tauopathies in the Elaboration of Therapies

Three main observations on hyperphosphorylation of tau during brain development and hibernation could impact the elaboration of therapies to prevent tau pathology and/or slow down its propagation in the brain. Firstly, the comparison of tau phosphorylation sites between physiological conditions and tauopathies indicates that the combination of phosphorylation sites is most likely a determinant factor in the toxicity mediated by hyperphosphorylated tau and therefore this should be taking into consideration when designing tools to neutralize phosphorylated forms at specific sites. Secondly, tau hyperphosphorylation is not found in glial cells in physiological conditions as noted in primary tauopathies indicating that they could play important role in the process of neurodegeneration. Thirdly, all tauopathies are linked to aging.

### Therapies Targeting Tau Phosphorylation Sites

Examining tau hyperphosphorylation in brain development and hibernation revealed that some sites could be protective preventing tau from forming toxic seed-competent aggregates. However, it is important to mention that a full characterization of tau phosphorylation sites using an approach such as MS is needed to identify the key sites involved in the shift from non-toxic to toxic tau species. Based on what it is known so far, it is clear that the combination of phosphorylation sites is a determinant factor in tau aggregation propensity. For example, in a recent study, it was reported that the amount of phosphorylation on doubly phosphorylated T231 and S235 and on singly phosphorylated S262, positively correlated with seeding capacity of tau whereas some phosphorylation sites negatively correlate with tau seeding such as the single phosphorylation within the clusters of sites contained in peptides S198/S199/S202 or S400/T403/S404 (133). This indicates that phosphorylation at specific sites likely results in conformational changes impacting tau seeding activity. Tau phosphorylation on tyrosine residues can also impact tau aggregation capacity. It was recently reported that tau phosphorylation at tyrosine (Y)18, Y29, Y197 at the N-terminal or at Y310 is sufficient to reduce tau aggregation (134). NMR indicates that there is a local decrease of beta-sheet propensity of tau's PHF6 domain when these sites are phosphorylated. In contrast, Briner et al. showed that the single phosphorylation at Y18 increases tau aggregation *in vitro* in presence of heparin (135). Lastly, tau phosphorylation at T205 was shown to be protective against Aβ in transgenic mice (136). From the above observations, one can conclude that specific pattern of tau phosphorylation can be either beneficial or detrimental to neurons and therefore to merely decrease global tau phosphorylation could result in undesired effects compromising neuronal survival.

### Glial Cells: An Unexplored Target

**Figure 2:**
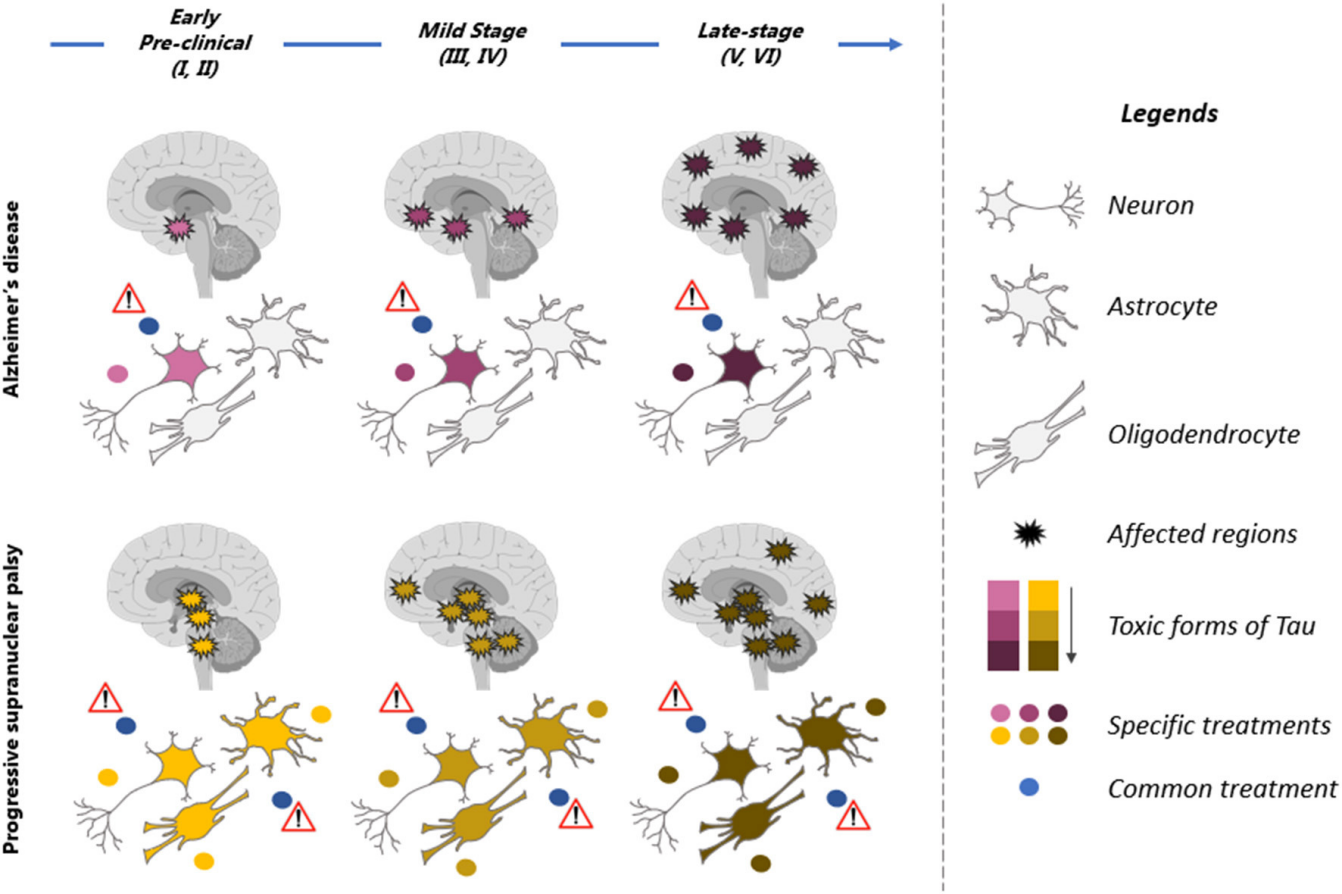
Therapeutic strategies for prevention of tau pathology in tauopathies. Illustration of the spatiotemporal pattern of the propagation of tau pathology in Alzheimer's disease (AD) and Progressive supranuclear palsy (PSP). Six stages (I–VI) of tau pathology can be observed in both diseases (top blue arrow). The affected regions in the brain are indicated by spiky circles. Tau pathology begins in distinct regions in each of these tauopathies and propagates differently. Tau pathology is predominant in neurons in AD brain while neurons and glial cells are affected in PSP. Different toxic forms of tau are observed during the course of the disease. They present a low toxicity (pink in AD and yellow in PSP) at early stages of the disease and high toxicity (dark red in AD and brown in PSP) at late stages of the disease. These forms are distinct in AD (yellow to brown) and PSP (pink to dark red). A therapeutic molecule should be designed for each toxic form of tau (yellow, brown, pink, and dark red dots). The therapeutic strategy should also be adapted to the stages of disease as tau forms vary during the course of the disease. Because the toxic forms of tau differs in AD and PSP, a molecule not specific to these forms (blue dot, common treatment) might be inefficient and even detrimental to patients by affecting forms of tau that are not toxic.

In some tauopathies, both neurons and glial cells are affected (Figure 2). It is still unclear if the increase of tau in glial cells is caused by dysfunction of the degradative mechanisms and/or the uptake of tau released by neurons. In PSP, tau pathology develops earlier in glial cells than in neurons in the globus pallidus and striatum (34). Hyperphosphorylated tau is not found in glial cells in developing brain and hibernation indicating that tau accumulation in these cells represents a pathological condition. Consistent with this, tauopathies developing tau pathology in glial cells such as PSP and CBD present a disease progression faster than AD where tau pathology is mainly found in neurons. In a recent study, it was reported that the uptake of extracellular tau oligomers by astrocytes, altered the intracellular Ca2+ signaling and Ca2+-dependent release of gliotransmitters resulting in synaptic dysfunction (137). Glial cells could also contribute the propagation of tau pathology in the brain. It was reported that microglia can facilitate tau pathology propagation between neurons by phagocytosing and exocytosing tau protein (138). Astrocytes were shown to decrease the spreading of tau pathology by taken up extracellular tau and targeting it for degradation in a mouse model (139). However, in PSP and CBD, the tau taken up by astrocytes might be exocytosed instead of degraded allowing the propagation of tau pathology in the brain.

**Figure 3:**
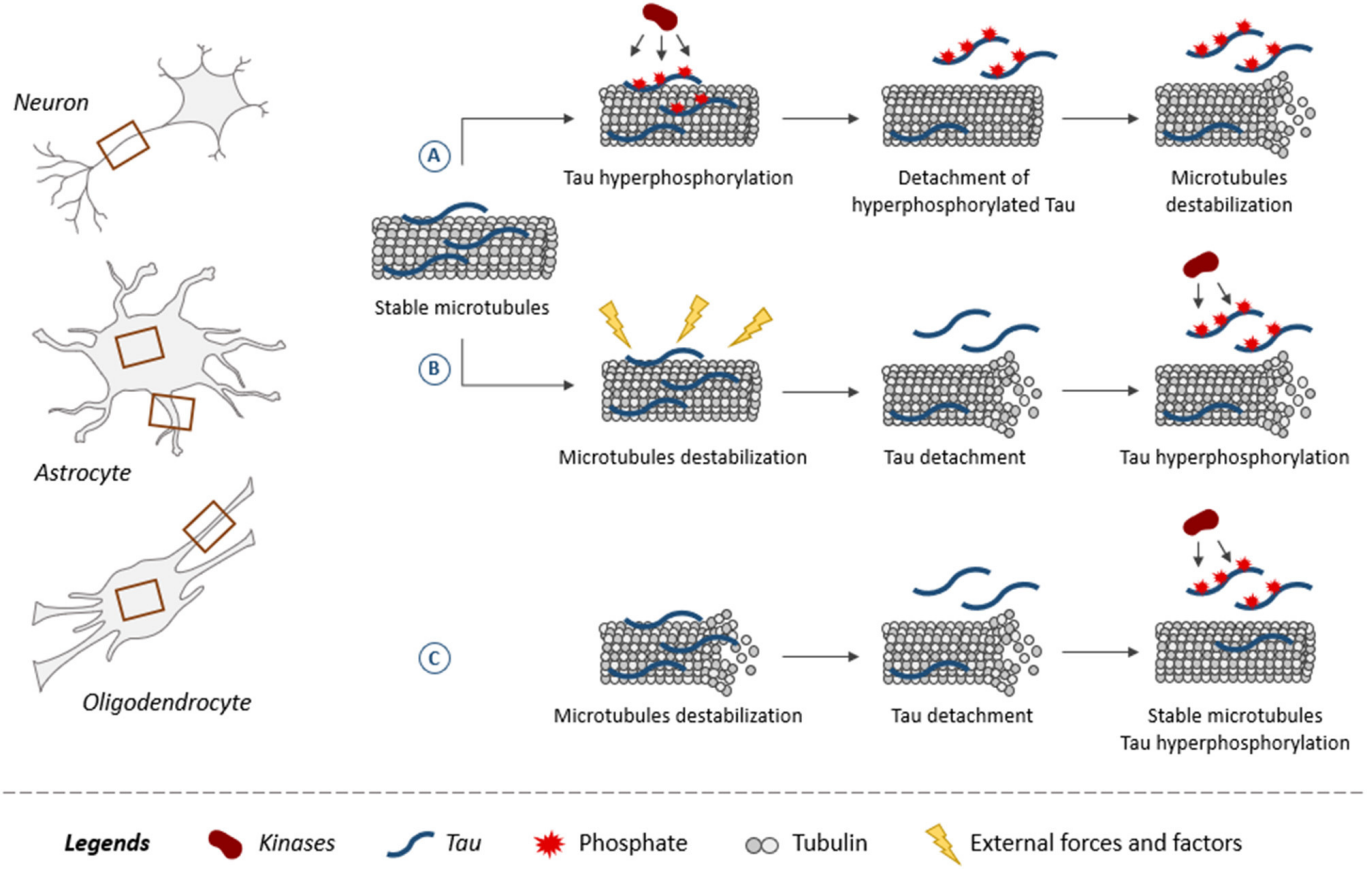
Role of tau and its phosphorylation in microtubule dynamics. Microtubules, composed of tubulin, are found in both neuronal and glial processes (astrocytes and oligodendrocytes) as indicated by brown squares. Illustration of three scenarios on how tau plays a role in microtubule dynamics within neurons and glial cells. Tau promotes assembly and stabilization of microtubules **(A,B)**. **(A)** When tau becomes hyperphosphorylated, it reduces its binding to microtubules resulting in its detachment. Microtubules are destabilized upon the loss of tau at their surface. **(B)** External forces and factors destabilize microtubules, leading to the detachment of tau increasing its access to kinases, which results in its hyperphosphorylation. **(C)** Tau binding does not stabilize microtubules but instead increases their dynamics. The detachment of tau from microtubules and its hyperphosphorylation is associated with an increase of microtubule stabilization.

The fact that glial cells are affected earlier than neurons in some brain regions could indicate that the insults triggering the disease altered the functioning of glial cells more than that of neurons in these regions. The mechanisms leading to tau pathology in glial cells remain poorly characterized. As shown for neurons, tau is believed to be attached to microtubules and to regulate their assembly in glial cells (140). This is supported by studies reporting that the overexpression of human tau in primary rat astrocytes induced selective destruction of detyrosinated microtubules while in oligodendrocytes, alterations of microtubule network were noted (141, 142). From this, one can speculate that microtubules might be a key element in the development of tau pathology in both neurons and glial cells. The most popular hypothesis is that tau becomes hyperphosphorylated and detaches from microtubules decreasing their stability (Figure 3) (12). However, the opposite could also occur, meaning microtubules become unstable and this weakens the binding of tau favoring its detachment. Indeed, microtubules are very sensitive to external forces and factors modulating cellular functions. Mutations in the tubulin gene are linked to severe developmental neurological disorders called tubulinopathies (143). Microtubules present in both neurons and glial cells might be involved in the initial phase of the disease. Under normal conditions, alternating cycles of phosphorylation and dephosphorylation regulate tau binding to microtubules. These cycles could be altered in tauopathies leading to tau hyperphosphorylation (144). Another possibility could be that during early stages of tauopathies presenting glial tau pathology, glial cells can proliferate involving the depolymerization of microtubules during this process. This depolymerization would result in tau detachment from microtubules and thereby its access to kinases would be increased as well as its phosphorylation levels. At late stages of AD, it was reported that astrocytes become senescent implying that they stop to divide (145). In such a cellular context, tau binding to microtubules could be blocked making irreversible its detachment from microtubules and its hyperphosphorylation state.

### Targeting the Aging Process

The fact that tau hyperphosphorylation does not exert any toxic effects during brain development and hibernation could indicate that the cellular context plays a pivotal role in determining whether hyperphosphorylated tau is part of either a response supporting neuronal survival or a response compromising neuronal survival. Four of the main changes occurring in aging could contribute to tau hyperphosphorylation. Firstly, it is well-documented that the efficacy of the two systems involved in tau degradation, the proteasome and the autophagy, is significantly decreased in aging favoring the accumulation of phosphorylated tau (146). Secondly, the combination of a decrease of energy supply and a decrease of clearance of damaged organelles could contribute to the triggering of tau pathology in aging. For example, the accumulation of dysfunctional mitochondria could restraint the supply of energy in aged cells (147). The inhibitors of the mitochondrial complex I, rotenone and annonacin, were shown to induce tau pathology in rat striatal neurons and glial cells indicating that mitochondrial dysfunction in aging could favor tau pathology (148, 149). Thirdly, changes leading to a progressive increase in vascular resistance and decrease in tissue levels of oxygen and glucose are noted in aging (150). Chronic hypoxia was shown to induce tau hyperphosphorylation in a rat model (151). Fourthly, aging could favor the propagation of tau pathology in the brain. A decrease of glymphatic system, the brain's metabolite clearance system connected to the peripheral lymphatic system is impaired in aging (152). This system was recently shown to contribute to the clearance of extracellular tau from the brain, the pool of tau believed to be involved in the propagation of tau pathology in the brain (153).

## Section (IV) Hyperphosphorylated Tau: Direct or Indirect Therapeutic Target

Several clinical trials that directly target tau are currently active. The main ones are testing tau immunotherapy for sequestration of pathological tau species (154). Such an approach allows to specifically either target a phosphorylation epitope, a conformational state or a peptidic sequence of tau (155, 156). The epitope S396/S404 was reported to be an efficient therapeutic target. Two vaccines targeting this epitope, one active (ACI-35, ISRCTN13033912) and one passive (Lu AF87908, NCT04149860) are presently in clinical trials (154). An antibody directed against cis-phosphorylated T231 (NCT04096287) is also tested while the vaccine targeting S422 has been discontinued (157). Phosphorylation of tau at certain sites was solely noted in seed competent tau fractions isolated from AD brain (133). More precise identification of the sites involved in the conversion of non-competent to competent seed forms is needed to develop antibodies that could prevent formation of competent seed forms. Epitopes may not be accessible on the majority of tau seeds and therefore an analysis of the atomic structure of such tau species is needed to reveal the epitopes that could be easily accessed by an antibody. Furthermore, one group reported that preventing tau seeding and blocking tau toxicity are not necessarily linked. This was shown using an antibody directed against phosphorylated T212/S214 epitope that could prevent tau seeding but not tau toxicity indicating that seeding could be neuroprotective (158). Another aspect worthy of consideration is that some antibodies only act on extracellular tau to refrain the spreading of tau (159). The characterization of tau phosphorylation sites in CSF by MS has revealed that tau phosphorylation varies depending on the evolution stages of the disease (160). In such a case, different antibodies will have to be used to neutralize these tau species during the progression of the disease.

Phosphorylation epitopes that are shared between the tauopathies could be used to design antibodies that should work for all tauopathies unless these epitopes are rendered inaccessible to antibodies because of tau conformation. Indeed, the analysis of tau atomic structure using Cryo-EM has revealed that tau filaments are distinct in AD, PiID and CBD (24). These analyses revealed that some phosphorylation sites could dictate which tauopathie will develop. Falcon et al. (26) reported that Pick body filaments are not phosphorylated at S262/or S356 and proposed that S262 could be protective against PiD (26). A similar conclusion was found for other post-translational modifications. Using Cryo-EM, it was also shown that deamidation of asparagine (N) 279 was present in AD but not in CBD or PSP indicating that as noted for phosphorylation, this modification can dictate the type of disease that will develop (27). This indicates that when targeting either a specific phosphorylation site or any another post-translational modification site, it will be important not to induce a shift from one disease to another. Cryo-EM analysis of hyperphosphorylated tau aggregates in developing brain and in hibernating animals could help to identify the key sites preventing the formation of toxic tau species. Tau conformation might also change throughout the disease evolution, and the affinity of the antibody for a specific conformation as well reducing its affinity for tau (Figure 2) (156).

In several clinical trials, hyperphosphorylated tau is an indirect target. In most of these trials, either the inhibition of tau kinases or the activation of PP2A, the main tau phosphatase is tested (161). Tau is phosphorylated by several proline-directed and non-proline-directed kinases (4). One can expect that the inhibition of a sole kinase might not be sufficient to completely prevent tau hyperphosphorylation. Lithium carbonate, a GSK-3 inhibitor, (NCT0318528) and Nilotinb, an inhibitor of Tyrosine kinase Ab1 (NCT02947893) are presently in Phase IV and Phase II, respectively. The phosphatase PP2A can directly and indirectly by regulating the activity of tau kinases modulate tau phosphorylation (162). PP2A activity is decreased in AD brain and therefore several therapeutic strategies have been elaborated to increase its activity (161). PP2A activity can be increased by inhibition of its endogenous inhibitors, ${\text{I}}_{1}^{\text{PP}2\text{A}}$ and ${\text{I}}_{2}^{\text{PP}2\text{A}}$ (163, 164). PP2A activity can also be increased by preventing its inhibitory demethylation by methyl transferase or phosphotryosinylation at T307 by src kinase (165, 166). Compounds that can increase PP2A activity such as Metformin (NCT01965756), an anti-diabetic drug, and sodium selenite (ACTRN12611001200976), which enhance PP2A activity by promoting the stabilization of its catalytic subunit PP2Ac, are currently in Phase II. So far, the same sites were found to be phosphorylated in neurons and glial cells (34). This could signify that the kinases and phosphatases involved in tau hyperphosphorylation are shared by neurons and glial cells and therefore any strategy targeting either tau kinases or phosphatases should work in both neurons and glial cells in tauopathies where these two types of cells are affected.

Tau phosphorylation can be regulated by other post-translational modifications. O-GlcNAcylation was shown to regulate tau phosphorylation (167). In AD brain, the decrease of O-GlcNAcylation of tau was correlated to an increase of tau phosphorylation (168, 169). The inhibition of O-GlcNAcase, the enzyme that removes the O-GlcNAc moieties is tested as an approach to decrease tau phosphorylation. Such inhibitors (AsceNeuron) are presently in Phase I. However, since 100 of proteins undergo O-GlcNAcylation, secondary effects could be triggered by these inhibitors. In a recent study, it was demonstrated that HDAC6 reduce tau acetylation and phosphorylation in the microtubule-binding domain (170). Its suppression in transgenic mice reduces their survival correlated to accelerated tau pathology and cognitive decline revealing HDAC6 as a potential therapeutic target.

At early stages of the disease, tau detachment from microtubules in both neurons and glial cells could be reversible and therefore therapy aiming at increasing tau binding to microtubules could be efficient in stopping the disease. On one hand, if tau first detaches from microtubules because of its hyperphosphorylation, an effective therapy would be to prevent this hyperphosphorylation. On the other hand, if changes in microtubule dynamics trigger the detachment of tau from them, the therapy should act on microtubules instead of tau. This avenue was explored by using drugs that stabilize microtubules. Promising results were obtained in tau mouse models but no beneficial effect was observed in humans (171–173). This could be explained by the fact that a recent study reported that tau does not stabilize microtubules but exerts the opposite effect (Figure 3). Indeed, the suppression of tau was associated with an increase of microtubule stabilization in rat cortical neurons (174). The controversy on the stabilizing effects of tau on microtubule needs to be resolved before the elaboration of an efficient therapy.

The interactome of hyperphosphorylated tau was recently characterized (175). In particular, the interactome of phosphorylated tau found in NFTs microdissected from patients with advanced AD was compared to that of phosphorylated tau immunopurified with the PHF-1 (S396/S404) antibody. Seventy-five proteins of the 125 proteins interacting with immunopurified tau were present in NFTs. These proteins were involved in phagosome maturation, the regulation of synaptic plasticity, DNA binding proteins and members of the 14-3-3 family. From these data, it is reasonable to speculate that these interactions are modulated by tau phosphorylation state and therefore could be modulated during the progression of the disease. The manipulation of tau phosphorylation by any treatment should take this into account for not favoring interactions that are detrimental to neurons.

Lastly, a recent study revealed that excitatory neurons having a selective vulnerability to tau pathology present an elevated expression of tau aggregation-prone proteins and a decreased expression of tau aggregation protector proteins (176). This indicates that a therapy targeting one of these groups of proteins could be beneficial for all tauopathies. Furthermore, these proteins were higher in neurons than in glial cells explaining why neurons are more affected by tau pathology than glial cells in the most frequent tauopathies (176). However, in tauopathies where glial cells are affected before neurons and in tauopathies where only glial cells are affected, the therapy developed for preventing tau pathology in neurons might not be efficient unless it is demonstrated that the same proteins favoring tau aggregation or aggregation protector proteins can play similar roles in glial cells.

From the above observations, one can speculate that therapies targeting mechanisms involved in tau hyperphosphorylation such as the inhibition of tau kinases would be efficient in all tauopathies but a lot more complex to elaborate because of their secondary effects on substrates other than tau. Using tau as a direct target appears to be the most achievable approach although the diversity of pathological tau forms implies the adaptation of a therapy for a each tauopathy. The success of the direct approach relies on a full characterization of tau phosphorylation sites and its other post-translational modifications in both physiological and pathological conditions to identify the pivotal sites in the conversion from non-toxic to toxic species. Furthermore, as revealed in a recent study, tau phosphorylation varies during the progression of AD indicating that the strategy applied at early stages of the disease will be different from that applied at later stages.

## Author Contributions

AV, CP, AD, and NL wrote the manuscript. AV prepared Tables 1, 2. CP prepared Figures 1–3. All authors contributed to the article and approved the submitted version.

## Conflict of Interest

The authors declare that the research was conducted in the absence of any commercial or financial relationships that could be construed as a potential conflict of interest.
